# A Rapid Cognitive and Motor Decline: A Case of Misdiagnosed Sporadic Creutzfeldt-Jakob Disease

**DOI:** 10.7759/cureus.73928

**Published:** 2024-11-18

**Authors:** Amrit Samra, Tasneem Ahmed, Mariya Ahmed, Taha Elsahy

**Affiliations:** 1 Acute Medicine, Peterborough City Hospital, Peterborough, GBR

**Keywords:** creutzfeldt jakob disease, misdiagnosis, neuropsychiatry, neuroradiology, palliative and supportive care, prion diseases, rapid cognitive impairment, rare neurological conditions, rt quic, stroke

## Abstract

Creutzfeldt-Jakob disease (CJD) is a fatal neurodegenerative disorder that leads to rapid cognitive decline, dementia, and neurological deterioration. CJD has several forms, including sporadic CJD (sCJD), which accounts for most cases, and variant CJD (vCJD), linked to exposure to bovine spongiform encephalopathy (BSE or mad cow disease). The disease is caused by abnormal prion proteins, which damage the brain and lead to death. CJD is universally fatal, with no cure, and most cases are fatal within a few months to a year after diagnosis.

We present a 64-year-old woman with a four-week history of progressive yet sudden decline in cognitive function and memory loss. There was associated decline in motor function, slumped posture, and an unsteady gait. Key findings included rapid cognitive impairment, apraxia, ataxia, hyperreflexia, and hypertonia. After extensive diagnostic examinations, including magnetic resonance imaging (MRI) head and cerebrospinal fluid real-time quaking-induced conversion (CSF RT-QuIC), she was diagnosed with sporadic Creutzfeldt-Jakob disease using criteria set by the national CJD research and surveillance unit (NCJDRSU). MRI head showed multifocal cortical ribboning on diffusion-weighted imaging and borderline right caudate hyperintensity. Unfortunately, there is no cure for CJD, and treatment is purely supportive with palliative care input, family counseling, and anticipatory medications. Her symptoms worsened quickly, and she passed away eight weeks after the onset.

## Introduction

Creutzfeldt-Jakob disease (CJD) is a rare, universally fatal neurodegenerative disorder caused by abnormal folding of the prion protein, resulting in the progressive accumulation of misfolded proteins within the brain [1]. With a global incidence of 1-2 cases per million, CJD primarily occurs sporadically (85-90% of cases), though familial, iatrogenic, and variant forms also exist [2]. Sporadic CJD (sCJD), the most common form, generally affects individuals around age 65, with most cases appearing between the ages of 60 and 70, although it has been documented across a wide age range [3,4]. The disease’s underlying cause remains unclear, and it impacts both genders almost equally [2]. Early symptoms of CJD vary but often begin with cognitive impairment in about 35% of patients, followed by cerebellar dysfunction, behavioral disturbances, or constitutional symptoms in others [5]. The prognosis is extremely poor; most patients succumb within a year of symptom onset. As the disease advances, patients may experience severe neurological deficits, including blindness, loss of speech, heightened susceptibility to infections due to immobility, and, in later stages, may enter a comatose state. The major cause of death is often infections, such as pneumonia, as the patient's immune system becomes severely compromised.

This report details a 64-year-old woman who presented with rapid neurological decline initially misattributed to stroke, later evaluated with MRI and CSF testing. The radiological findings raised several differential diagnoses, with CJD emerging as a key consideration. The patient’s clinical and imaging findings met the national CJD research and surveillance unit’s (NCJDRSU) diagnostic criteria, supporting a probable CJD diagnosis. This prompted CSF analysis using real-time quaking-induced conversion (RT-QuIC) testing, which confirmed the presence of abnormal prion proteins and the diagnosis of sCJD. Given its rarity and the critical role of early, accurate detection, this case underscores the importance of clinical vigilance and comprehensive diagnostic workup in suspected CJD.

## Case presentation

Medical history and demographics

The patient is a 64-year-old White British female, retired from a career in administration, with a history of hypertension. She also had chronic insomnia, previously managed with zopiclone several years ago. She presented with a progressive decline in cognitive and motor function, which was first noticed by her husband over 4 weeks Initially, her husband observed a reduction in her ability to manage finances, specifically struggling with tasks she previously handled with ease, such as tracking expenses on a spreadsheet. This cognitive decline was accompanied by progressive memory loss, manifesting in her forgetting simple tasks, such as dates, or leaving household items like the tap running. In one notable instance, she mistakenly placed fabric conditioner in a pot instead of the washing machine. 

In addition to the cognitive symptoms, the patient began to experience a decline in motor function. She found it increasingly difficult to walk up the stairs, requiring support from the banister, and her posture became more slumped. She also had difficulty maintaining a straight gait. These changes were especially noticeable to her son, who had seen her fit and well just a month prior. This prompted a visit to the emergency department. At her initial hospital presentation, routine blood tests were normal, and a CT head scan was unremarkable. She was discharged without a clear diagnosis. However, following ongoing concerns from the family, she reattended the hospital the very next day. 

At the second presentation, the primary differential was an acute stroke. On examination, the patient exhibited an unsteady gait and signs of left-sided neglect. Visual fields were full to finger counting, but there was evidence of left-sided visual neglect. There was increased tone (paratonia) and brisk reflexes, particularly in the left limbs. Finger-nose ataxia was present on the left side, and she demonstrated difficulty performing complex hand movements, suggesting apraxia. Additionally, she was disoriented, unable to recall her symptoms, and unaware of her hospital admission. She believed the year was 1964 and was not oriented to time or place. There was no history of seizures, collapse, toxin exposure, hypoxia, or any prior brain or eye surgeries. The patient had not received any blood transfusions or blood products, though she had donated blood around 20 years prior. She consumed minimal alcohol, did not smoke, and did not use recreational drugs or self-medicate. The family noted that she had been under significant personal stress recently, particularly in relation to organising care for her elderly parents.

Investigations

**Figure 1:**
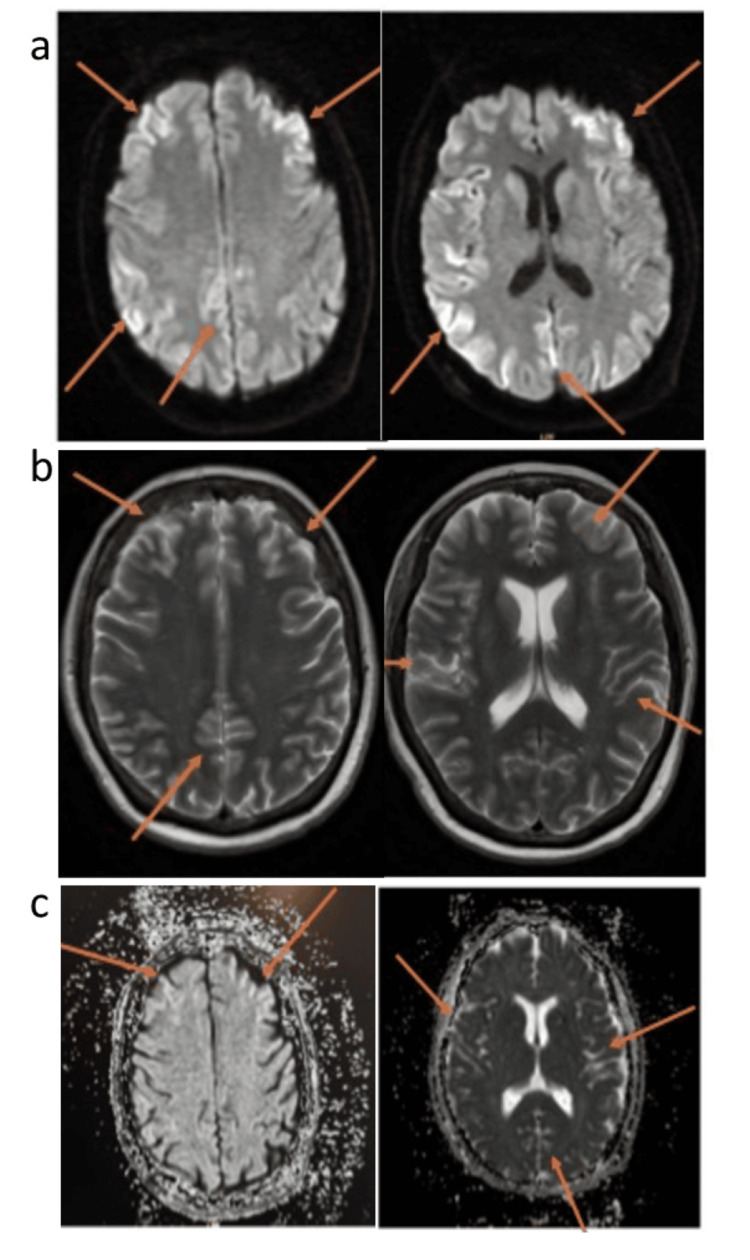
Bilateral cerebral hemispheres almost symmetrical cortical diffusion restriction, showing; a) high signal on DWI, b) low signal on ADC map and c) slightly increased signal on T2/FLAIR sequence DWI: diffusion-weighted imaging, T2/FLAIR: T2-weighted imaging/fluid-attenuated inversion recovery, ADC: apparent diffusion coefficient.

Initial blood tests showed normal full blood count, renal function, electrolytes, haematinics, and inflammatory markers, with the only abnormality being a mildly elevated Alanine aminotransferase (ALT). The CT head scan did not demonstrate any significant findings. Following the stroke protocol, an MRI was performed, which demonstrated bilateral cerebral cortical diffusion restriction and mild increased Fluid-Attenuated Inversion Recovery signal intensity (T2/FLAIR) (Figure 1). 

Based on the MRI findings, the radiologist noted a broad differential diagnosis, including hypoxic-ischemic encephalopathy, toxic/metabolic encephalopathy, or encephalitis, with CJD as a possibility. An urgent neurology review was recommended. After neurologist consultation, given the patient's rapid cognitive and motor decline and no obvious toxic or metabolic cause, sCJD became the leading differential. A cerebrospinal fluid (CSF) sample was obtained for RT-QuIC testing. The family was counseled about the possibility of CJD, preparing them for its implications. The CSF results revealed a normal cell count but tested positive for RT-QuIC, confirming the diagnosis of sCJD. 

Outcomes

Based on the rapidly worsening cognitive and motor decline over the space of one month, with correlating imaging and a CSF sample detecting pathological prion proteins, the diagnosis was established as sCJD.  The patient was transferred to the infectious disease ward, where staff and family initially wore personal protective equipment (PPE). However, after consultation with the neurology team from the national CJD research and surveillance unit in Edinburgh, it was clarified that sCJD is not contagious under normal circumstances. This guidance allowed the patient’s family and friends to visit her without restriction, providing much-needed physical closeness and support as she neared the end of life. The palliative care team became involved in prioritizing the patient’s comfort and managing her symptoms with opioids like morphine for pain relief, benzodiazepines for muscle spasms and myoclonus, and sedatives and antidepressants for severe agitation and anxiety. Non-pharmacological measures supported the patient and her family, including assistance with daily activities (eating, hygiene, mobility), infection monitoring, and emotional support. To maintain her dignity, she was provided privacy in a side room. The overall goal of palliative care in CJD was to ensure symptom relief, comfort, and quality of life while supporting her family through the disease's progression. Over the following weeks, the patient’s condition worsened, with increasing memory loss, withdrawal, and reduced mobility. She then entered a comatose state with reduced responsiveness and, despite efforts to support her, passed away from respiratory failure within four weeks of admission.

## Discussion

CJD is a rare, rapidly progressing neurodegenerative disorder that poses significant diagnostic challenges due to its subtle early symptoms. This case highlights a patient initially misdiagnosed with a stroke despite early neurological imaging showing no abnormalities. The delay in diagnosis emphasizes the importance of maintaining a broad differential when faced with unexplained cognitive and motor decline. The patient's rapid deterioration, without clear toxic or metabolic causes, complicated the diagnosis. This case underscores the challenges in diagnosing sCJD, particularly when initial symptoms are psychiatric in nature. The patient, under considerable stress following a family bereavement and with a longstanding history of insomnia, could have been misdiagnosed as having purely psychiatric issues. This case stresses the need for heightened clinical vigilance, especially when psychiatric manifestations may mask underlying neurodegenerative disease [6].

Diagnosis

During the stroke evaluation, the patient underwent an MRI, which showed bilateral cerebral cortical diffusion restriction and mild increased T2/FLAIR signal intensity. These findings raised a broad differential diagnosis, including hypoxic-ischemic encephalopathy, toxic/metabolic encephalopathy, encephalitis, and CJD. The diffusion restriction was particularly significant, suggesting prion-related damage in cortical and subcortical regions, commonly seen in CJD [7,8]. RT-QuIC testing, a highly sensitive and specific diagnostic tool for sporadic CJD, was performed [9]. This test detects pathogenic prion proteins by using a recombinant prion protein substrate that aggregates in their presence. The patient's positive RT-QuIC result confirmed the diagnosis of CJD. The current sensitivity of CSF RT-QuIC undertaken at the UK NCJDRSU is 92%, and the specificity is 100% [10]. This case highlights the critical role of advanced diagnostic methods like RT-QuIC in ensuring accurate and timely diagnosis of neurodegenerative disorders, enabling appropriate management and support for patients with CJD (Tables 1-2). 

**Table 1:** NCJDRSU criteria for sporadic CJD EEG: electroencephalogram; DWI: diffusion-weighted imaging; FLAIR: fluid-attenuated inversion recovery.

I	Rapidly progressive cognitive impairment
II	(1) Myoclonus; (2) Visual or cerebellar problems; (3) Pyramidal or extrapyramidal features; (4) Akinetic mutism
III	Typical EEG (generalized periodic complexes)
IV	High signal in caudate/putamen on MRI brain scan or at least two cortical regions (temporal, parietal, occipital) either on DWI or FLAIR

**Table 2:** Diagnosis method for NCJDRSU criteria for sporadic CJD NCJDRSU: national CJD research and surveillance unit [11], CJD: Creutzfeldt-Jakob disease I, II, III, IV are criteria listed in Table 1.

Criteria	Definite	Probable	Possible
Diagnosis method	Progressive neurological syndrome and neuropathologically or immunocytochemically or biochemically confirmed	(a) I+2 of II and III; (b) I+2 of II and IV; (c) I+2 of II and positive I4-3-3; (d) progressive neurological syndrome and positive RT-QuIC in CSF or other tissues	I+2 of II+duration <2 years

Treatment 

CJD has no known cure, and treatment focuses on symptom management and patient comfort. Palliative care is the primary approach, with options like hospice or specialized care homes recommended to provide appropriate support. Ongoing research continues to explore potential therapeutic options for CJD [12].

Latest updates on CJD

Recent research into CJD has focused on understanding the prions involved in both human and animal forms of the disease. These studies aim to identify factors that affect prion infectivity and how they lead to brain damage. Key areas of investigation include the mechanisms of abnormal prion formation and accumulation and how prions replicate within specific brain cells. Additionally, researchers are exploring how prions cross the blood-brain barrier and spread throughout the central nervous system. Findings from these studies may eventually help guide therapeutic strategies for managing prion diseases [13]. 

While tissue testing remains the gold standard for confirming a diagnosis of CJD, it is important to recognize that biopsy results can sometimes yield inconclusive findings. A robust clinical suspicion is essential for proceeding with a biopsy, as a negative result does not definitively exclude a diagnosis of CJD; the pathology may be localized to specific regions of the brain. Consequently, a biopsy is often unlikely in such scenarios [14].

## Conclusions

In conclusion, this case underscores the complexity and diagnostic challenges of CJD, particularly its sporadic form. The patient’s initial presentation with subtle cognitive and behavioral changes, compounded by a stressful life event and insomnia, masked the early signs of a neurodegenerative disorder. Despite the non-specific findings on her initial CT and MRI, clinical suspicion remained high. The definitive diagnosis was supported by the positive RT-QuIC test, which confirmed the presence of pathological prion proteins. This case highlights the necessity of considering CJD in patients with rapid cognitive decline and the importance of timely, comprehensive evaluations. It also emphasizes the need for heightened awareness, particularly when initial diagnostics do not provide clear answers, to avoid misdiagnosis and delayed treatment in this devastating condition.
